# glbase: a framework for combining, analyzing and displaying heterogeneous genomic and high-throughput sequencing data

**DOI:** 10.1186/2045-9769-3-1

**Published:** 2014-01-24

**Authors:** Andrew Paul Hutchins, Ralf Jauch, Mateusz Dyla, Diego Miranda-Saavedra

**Affiliations:** 1 Key Laboratory of Regenerative Biology, South China Institute for Stem Cell Biology and Regenerative Medicine, Guangzhou Institutes of Biomedicine and Health, Chinese Academy of Sciences, Guangzhou, 510530 China; 2 Genome Regulation Laboratory, South China Institute for Stem Cell Biology and Regenerative Medicine, Guangzhou Institutes of Biomedicine and Health, Chinese Academy of Sciences, Guangzhou, 510530 China; 3 Laboratory for Structural Biochemistry, Genome Institute of Singapore, 60 Biopolis Street, Singapore, 138672 Singapore; 4 Department of Molecular Biology and Genetics, Aarhus University, Aarhus, Denmark; 5 Fibrosis Laboratories, Institute of Cellular Medicine, Newcastle University Medical School, Framlington Place, Newcastle upon Tyne, NE2 4HH UK

**Keywords:** ChIP-seq, RNA-seq, Genomics, Microarray, Motifs, Transcription factor, Bioinformatics

## Abstract

Genomic datasets and the tools to analyze them have proliferated at an astonishing rate. However, such tools are often poorly integrated with each other: each program typically produces its own custom output in a variety of non-standard file formats. Here we present glbase, a framework that uses a flexible set of descriptors that can quickly parse non-binary data files. glbase includes many functions to intersect two lists of data, including operations on genomic interval data and support for the efficient random access to huge genomic data files. Many glbase functions can produce graphical outputs, including scatter plots, heatmaps, boxplots and other common analytical displays of high-throughput data such as RNA-seq, ChIP-seq and microarray expression data. glbase is designed to rapidly bring biological data into a Python-based analytical environment to facilitate analysis and data processing. In summary, glbase is a flexible and multifunctional toolkit that allows the combination and analysis of high-throughput data (especially next-generation sequencing and genome-wide data), and which has been instrumental in the analysis of complex data sets. glbase is freely available at http://bitbucket.org/oaxiom/glbase/.

## Background

Genome-scale experiments are rapidly becoming a standard addition to the scientists’ toolkit. However, the development of tools to analyze high-throughput data has lagged behind our ability to generate larger and larger data-sets, and despite some standardization efforts, custom file formats continue to proliferate. Many of the tools currently used to analyze genome-wide data are very diverse and produce a variety of custom outputs that rarely feed directly into other bioinformatics tools without pre-processing of the file into standard file formats. A common way to get around this is to create *ad hoc* scripts in some combination of UNIX shell, awk, Perl, Python or other programming language and use these scripts to address the problem at hand. However, these scripts are often designed with only a single usage in mind, lack a detailed methodology, may be poorly documented or not preserved at all, and are rarely tested for accuracy and consistency.

Efforts have been made to make this process more transparent; Galaxy is a comprehensive web server with a large number of functions to deal with genome-scale data [1], but it is a web-server aimed primarily at non-programming scientists, requires extensive user interaction and therefore is difficult to automate, thus losing the advantages of a programming environment or the UNIX shell. BEDTools [2] and SAMtools [3] deal efficiently with the standardized genome file formats BED and SAM, but do not deal gracefully with non-standard file inputs or even poorly or incorrectly formatted files. The Biopython [4] and Bioperl [5] projects similarly attempt to deal with these problems, but these projects have such a large scope across all of their subject areas that the analysis of high-throughput sequencing has been relatively neglected to date.

**Figure 1:**
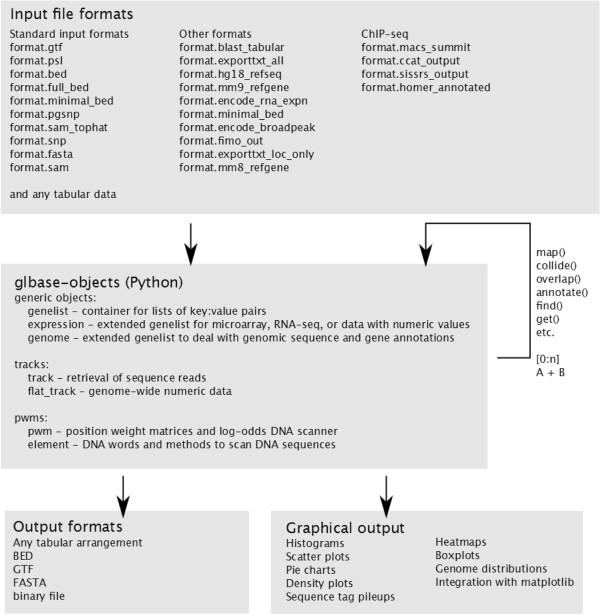
**A schematic overview of the functions included in glbase.** glbase accepts files in a variety of formats, brings them into a Python environment as ‘*genelist*’ objects which behave like a Python list of key:value pairs. Data can be manipulated within glbase using a variety of built-in functions, and subsequently output in specific formats or graphically for the visual interpretation of (combined) datasets.

The Bioconductor [6] project for the R language has a massive scope, with multiple tools from multiple developers that can come together to form a potent analysis toolkit. It is well documented and has become one of the major analytical frameworks for genomic analysis. Yet it has some limitations, the R language has a steep learning curve and deployment of a users own methods or functions is difficult. One of the original motivations for the development of glbase was to format files suitable for the import format required by R and it still fulfills this role. The Genomic Hyperbrowser [7] takes an interesting novel approach to the analysis of genomic data, built on top of the Galaxy framework it uses the widespread concept of ‘tracks’ (i.e. collections of genomic features, genes, exons, epigenetic data, etc) to which the user defines a putative relationship describing the two tracks and a null model and then the Hyperbrowser will test this relationship. In this way the Hyperbrowser brings a more statistical and mathematical approach to the analysis of genomic data. Although primarily presented as a web server it also makes available a programmatic interface. ArrayPlex [8] provides a framework similar to glbase for the analysis of heterogenous genomic data, in addition to providing a graphical interface it also exposes its functionality through the UNIX shell as executable commands. ArrayPlex is mainly focused on the retrieval of data from publicly accessible webservers. CruzDB [9] is the tool most similar to glbase. Also implemented in Python it provides a convenient system to extract data primarily from the UCSC genome browser, process the data in Python and then submit the data to other tools. It does not contain any internal drawing methods, although it should integrate well with Python plotting libraries such as matplotlib and potentially also with glbase. Tools originally designed for DNA motif discovery, such as HOMER [10] and MEME [11] are also expanding in their scope and offer an increasing diversity of genomic analysis methods that are exposed to the user not only in the form of a web server but also as tools that can integrate with the command line for automation.

glbase is a project designed to complement the above tools for the analysis of genomic data. Using the advantages of the Python programming language glbase aims to directly translate biological questions into Python code. To assist in that glbase deals with several problems. Firstly it acts as an intermediary between tools. Secondly it provides a relatively compact programming syntax. Thirdly it incorporates many common analytical methods to integrate data. Finally, glbase provides tools for the graphical output of data analyses. glbase deals with the problem of incompatible file formats between different tools not by suggesting a top-down standardization of file formats, but instead by providing a simple means to describe diverse file formats and load them into a Python programming environment. Additionally, glbase facilitates the down-stream processing of the data as it includes a suite of common analysis tools, such as heatmaps and sequence read pileups. glbase has been designed to interact more generally with other Python tools, such as statistics with SciPy and graphical outputs with matplotlib, and data can also be exported into other file formats for analysis in yet further tools or imported into R. In this way glbase acts as the ‘glue’ between up-stream analysis (e.g. the genomic alignment of sequencing reads and ChIP-seq peak discovery) and down-stream analysis (e.g. ChIP-seq peak annotation, combining ChIP-seq/RNA-seq data, and the production of publication-quality figures). glbase is implemented as a Python module designed to be used non-interactively to write short scripts to achieve specific aims, leaving a permanent record of the user’s processes, thus documenting the data analysis process to make it repeatable. Furthermore, glbase incorporates methods to overlap and annotate genomic intervals (similar to BEDTools [2]), to map common values across two lists (similar to but more powerful than the UNIX command ‘join’), support for genomic coordinates to gene annotations and for extracting sequence data from FASTA files. Also included in glbase is a selection of analysis tools to produce a variety of graphical summaries of data, including heatmaps, scatter plots, pie charts and histograms of genomic and expression data. Finally, glbase features a flexible and efficient SQL implementation for storing genomic-scale data, such as high-throughput sequence reads or phastCons evolutionary scores [12], which allow the efficient random-access retrieval of numerical or sequence reads from within millions of sequencing tags. Figure 1 gives a schematic overview of the functions available in glbase. glbase is especially suited to the analysis of next generation sequencing and genome-wide data, particularly ChIP-seq, RNA-seq and microarray expression data.

## Results and discussion

### *Genelists* and flexible file format specifiers

glbase is built primarily around objects called ‘*genelists*’ , which are lists of key:value pairs with many associated methods. For example, given the output from the MACS peak-discovery tool [13], here in the format of a BED file, it can be loaded using two lines of Python:


The contents of the *genelist* can be interrogated, showing the index, and a list of < key>:<value > pairs:


The *genelist* object behaves in a manner similar to a normal Python list and can be iterated over, and its values extracted, sorted, sliced and searched. In addition, *genelists* contain many special methods for working on genomic intervals, particularly for intersecting two lists of genomic locations (similar to BEDTools [2]), but does not require the files to be in BED format, only that they have a correctly formatted ‘loc’ key containing a genomic interval resembling ‘chr1:1000000-1001000’. Genomic intervals can be systematically modified:


*Genelists* can also be intersected by pairs of matching keys, made unique for any key, and many other methods to manipulate the data contained within the *genelist*. Finally, the resulting *genelists* can be saved in a variety of file formats, such as custom TSV (tab-separated value) and standard BED files.

### Flexible specifiers to describe any arrangement of tabular data

In addition to loading standard file formats, such as BED, SAM, GTF/GFF and FASTA, glbase includes a flexible way to describe any tabular file format (for example tab-separated value [TSV] and comma-separated value [CSV] files). glbase just needs to know the names of the keys and the column number they appear in inside the TSV to load the file into glbase. For example, this line of code will describe the full formal definition of a BED file:


In the example above each value specifies the key name and the column number of the TSV file to find the data in. This flexible format specifier can be used to describe almost any TSV file for loading into glbase.

### Analysis and graphical outputs

**Figure 2:**
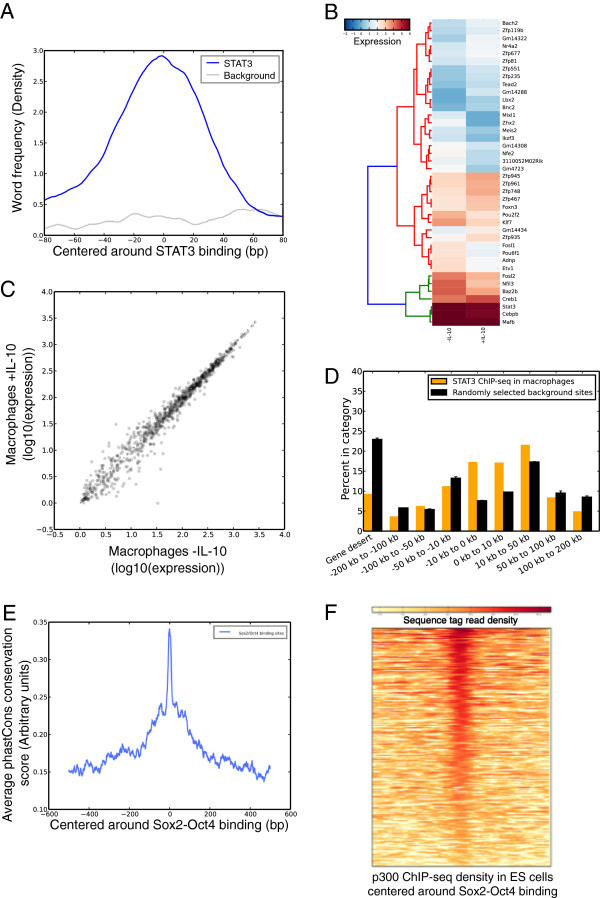
**Example graphical output from glbase.** Code and raw data can be found in the glbase directory (glbase/examples/). **(A)** Frequency of the STAT3 DNA-binding word (‘TTCnnnGAA’) in a list of STAT3 ChIP-seq binding sites, compared to a random selected background from the control ChIP-seq sample. **(B)** Heatmap of top 20 and bottom 20 up- and down-regulated transcription factors when macrophages are stimulated with IL-10. **(C)** Scatter plot of RNA-seq data **(D)** Genomic distribution of STAT3 binding in IL-10 stimulated macrophages. **(E)** Average phastCons evolutionary conservation score around a list of Sox2-Oct4 ChIP-seq binding peaks. **(F)** Heatmap of p300 recruitment in mouse ES cells for a list of Sox2-Oct4 ChIP-seq binding peaks. Raw data comes from the GEO accessions GSE31531 [14], the ENCODE project [16], GSE11431 [15] and the phastCons measure of evolutionary conservation [12]. Transcription factor annotation was based on the DNA-binding domain database [17].

In addition to acting as a universal file format converter, a second major utility of glbase is to act as the ‘glue’ between up-stream and down-stream analysis tools, for instance to get from a list of ChIP-seq peaks and gene expression values to heatmaps, gene-peak associations and other informative plots. As an example of usage, glbase includes a tool for finding words in FASTA-formatted DNA sequences: Figure 2A shows an example of the frequency of the STAT3 DNA-binding motif (word) ‘TTCnnnGAA’ in a list of STAT3 ChIP-seq binding data [14]. For any key in a *genelist*, its frequency can be measured with a pie chart. glbase can also deal with expression data through the derived *genelist*-like object ‘*expression*’ that contains methods for drawing heatmaps (Figure 2B) as well as histograms, boxplots, scatter plots (Figure 2C) and the ability to transform the expression data (fold-change, log-transform, normalize, etc.). Expression data and ChIP-seq data can be combined to produce density maps of ChIP-seq binding against changes in gene expression or to annotate scatter plots. ChIP-seq data can be compared against any set of genomic annotations, for example gene transcription start sites, to produce a breakdown of distances from the binding site to the transcription start site. Figure 2D shows the distribution of STAT3 binding sites in IL-10 stimulated macrophages relative to the nearest transcription start site [14]. Phylogenetic data (e.g. phastCons scores of evolutionary conservation, any type of numeric data can be used) can be loaded into an SQL database by glbase and then pileups can be visualized (Figure 2E). Similarly, sequence reads can be converted by glbase into an SQL database for efficient retrieval of the reads across arbitrary genomic locations. Figure 2F shows a heatmap of the density of sequence tag reads from a p300 ChIP-seq library centered on a list of Sox2-Oct4 bound region in embryonic stem cells [15, 16].

## Conclusions

glbase is a flexible and multifunctional toolkit allowing the user to perform many common analyses on ChIP-seq, microarray and RNA-seq data. Data from distinct sources can be combined inside a unified framework within a Python programming environment for direct analysis of the data, or processed and output for further analysis. glbase has already been used extensively in the analysis of STAT3 binding in macrophages [14], the analysis of STAT3 binding in multiple cell types [18], in analyzing the changes in the transcriptome of stimulated CD4^+^ T cells [19], and in the analysis of how mutated Sox17 co-operates with Oct4 to specify induced pluripotent stem cells [20, 21]. Thus glbase constitutes a useful addition to the researchers’ toolkit.

### Availability and requirements

glbase was developed in Python and uses the freely available Python modules NumPy, SciPy and matplotlib. All functions in glbase are documented in Python (for example, to see the documentation for the map() method of genelists, type: help(glbase.genelist.map)), and documentation is also available as part of the distribution (glbase/docs/build/html/index.html), which also includes seven tutorials, code and example raw data (glbase/examples/) directly aimed at potential users with little or no Python experience. glbase is freely available from http://bitbucket.org/oaxiom/glbase/.

## Abbreviations

TSV: Tab-separated value
BED: Browser extensible data
GTF: Gene transfer format
SAM: Sequence alignment/map.
